# Evaluating Staff Attitudes, Intentions, and Behaviors Related to Cyber Security in Large Australian Health Care Environments: Mixed Methods Study

**DOI:** 10.2196/48220

**Published:** 2023-10-04

**Authors:** Martin Dart, Mohiuddin Ahmed

**Affiliations:** 1 School of Science Edith Cowan University Joondalup Australia

**Keywords:** computer security, cyber security, surveys, governance, mixed methods, Australia, delivery of health care

## Abstract

**Background:**

Previous studies have identified that the effective management of cyber security in large health care environments is likely to be significantly impacted by human and social factors, as well as by technical controls. However, there have been limited attempts to confirm this by using measured and integrated studies to identify specific user motivations and behaviors that can be managed to achieve improved outcomes.

**Objective:**

This study aims to document and analyze survey and interview data from a diverse range of health care staff members, to determine the primary motivations and behaviors that influence their acceptance and application of cyber security messaging and controls. By identifying these issues, recommendations can be made to positively influence future cyber security governance in health care.

**Methods:**

An explanatory sequential mixed methods approach was undertaken to analyze quantitative data from a web-based staff survey (N=103), with a concurrent qualitative investigation applied to data gathered via in-depth staff interviews (N=9). Data from both stages of this methodology were mapped to descriptive variables based on a modified version of the Technology Acceptance Model (TAM; TAM2). After normalization, the quantitative data were verified and analyzed using descriptive statistics, distribution and linearity measures, and a bivariate correlation of the TAM variables to identify the Pearson coefficient (*r*) and significance (*P*) values. Finally, after confirming Cronbach α, the determinant score for multicollinearity, and the Kaiser-Meyer-Olkin measure, and applying the Bartlett test of sphericity (*χ*^2^), an exploratory factor analysis (EFA) was conducted to identify the primary factors with an eigenvalue (λ) >1.0. Comments captured during the qualitative interviews were coded using NVivo software (QSR International) to create an emic-to-etic understanding, which was subsequently integrated with the quantitative results to produce verified conclusions.

**Results:**

Using the explanatory sequential methodology, this study showed that the *perceived usefulness* of security controls emerged as the most significant factor influencing staff beliefs and behaviors. This variable represented 24% of all the variances measured in the EFA and was also the most common category identified across all coded interviews (281/692, 40.6%). The word frequency analysis showed that *systems*, *patients*, and *people* represented the top 3 recurring themes reported by the interviewees.

**Conclusions:**

To improve cyber security governance in large health care environments, efforts should be focused on demonstrating how confidentiality, integrity, availability, policies, and cloud or vendor-based controls (the main contributors of *usefulness* measured by the EFA) can directly improve outcomes for systems, staff, and patients. Further consideration also needs to be given to how clinicians should share data and collaborate on patient care, with tools and processes provided to support and manage data sharing securely and to achieve a consistent baseline of secure and normalized behaviors.

## Introduction

### Background

In reviewing the literature that investigates cyber security effectiveness in health care, a repeated problem emerges regarding a lack of research into how and why human factors are responsible for up to 85% of all data breaches or security incidents impacting the sector [1]. This is an important element to consider, as health care is repeatedly identified by the Office of the Australian Information Commissioner as the industry reporting the largest number of data breaches via its legislated reported regime [2,3], and many of these breaches regularly feature in media headlines [4-6], causing concern among the public. The recognition that technology alone is not enough to ensure effective security creates an opportunity for a more holistic approach, pursuant of more attentive and integrated user involvement. Such an ecosystem, where users actively help to ensure that data are not inappropriately disclosed or technical systems undermined, has come to be known as the *human firewall* [7-9].

### Literature Review

In their investigation of this symbiotic nexus between technology and sociology in health care, Jalali et al [10] undertook a comprehensive bibliographic analysis of existing research. The authors concluded that as most of their 472 verified sources originated from technically focused science fields, human and organizational aspects may be understudied. A similar conclusion was reached 13 years earlier by Williams [11], who surmised that research on the protection of medical data is often technically focused, which does not effectively address the people-driven behavioral aspects integral to effective information security. Finally, Warren and Leitch [12] identified that health care requires more than improved technical solutions, highlighting the need for security design methods that consider both the technical and social aspects of information security.

To address these concerns, this study undertakes a mixed methods investigation of a heterogeneous sample of employees working within large Australian health care providers (LAHPs) to identify specific motivational factors that influence their security behaviors and beliefs. This concept of considering multiple and potentially compounding behavioral drivers is based on the key pillar of Ajzen’s [13] seminal work on the Theory of Planned Behavior (TPB). This includes the idea that the intentions to perform behaviors can be predicted based on the individual’s attitude toward that behavior, the subjective norms that surround them, or their perception of certain behavioral controls.

Several authors have pioneered the use of mixed methods techniques to undertake studies investigating the aspects of this challenge, and elements of their techniques and findings inform this paper. Foundational work in this methodology was undertaken by Hofstede et al [14], who recognized that differences in organizational structure and control systems are likely to produce variances, or idiosyncrasies, within different strata of staff members. After studying multiple organizations, the authors concluded that localized cultures are influenced by common practices, symbols, heroes, and meaningful rituals.

A further enhancement of the mixed methods approach for measuring employee attitudes, incorporating an NVivo-centered word cluster and frequency analysis, was undertaken by Ho et al [15]. Although the techniques in their paper were shown to be effective, the focus on employee perceptions on leadership outside of health care was not directly relevant to the audience whom this paper seeks to engage.

An application of this approach to the health care industry (in Indonesia) was undertaken by Fauzi et al [1] using a range of surveying and analysis techniques. Focusing on assessing how workplace stress levels might influence staff attitudes toward cyber security, the authors concluded that workforce stratification, based on intersectional criteria, is worthy of further study. This is a specific aspect that this paper seeks to incorporate.

Kwan et al [16] undertook a detailed survey of health care information management governance in the state of Victoria, Australia, using a large survey instrument and a mixed methods descriptive approach. The authors identified limitations in staff knowledge of data breach techniques, and a prioritization of audit and compliance concerns. The fact that their study was small (n=36), and comprised only information management staff, limits the applicability of these findings for larger health care systems.

Yeng et al [17] conducted a detailed quantitative survey on health care workers in Ghana and also considered the concept of “the human firewall” combined with a human-centered motivational theory (TPB). The authors identified “useableness” as a theme that strongly influenced user security behaviors.

### Objective

Building on the work of these examples, this study sought to undertake a more comprehensive and integrated discovery of how these issues apply in large and specifically Australian health care environments.

## Methods

### Foundational Methodology: Technology Acceptance Model

When the Technology Acceptance Model (TAM) emerged in the 1980s from broader research into users’ willingness to accept or use new technology systems (the *productivity paradox* [18]), it focused on the 2 key drivers of *perceived usefulness* (“Will this application help me perform my job better?”) and *perceived ease of use* (“Even if this application is useful to me, is it easy and worth the effort for me to use?”) [19].

**Figure 1:**
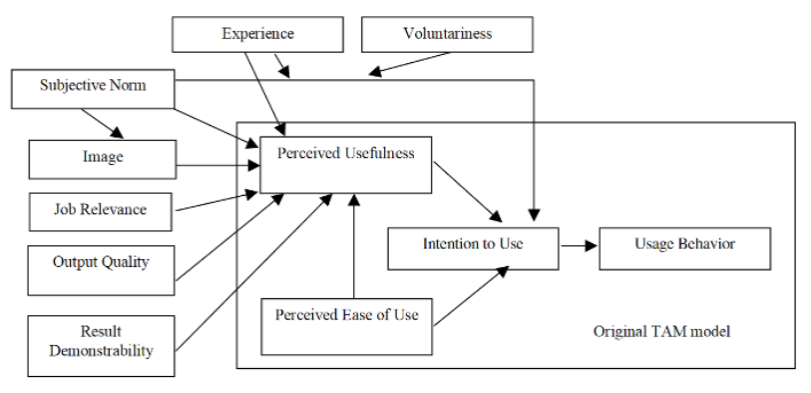
The original Technology Acceptance Model 2 (TAM2) model. ICT: information and communication technology, (reproduced from Venkatesh and Davis [20], with permission from The Institute for Operations Research and the Management Sciences [INFORMS)]).

TAM2 extends the TAM model by including 3 additional social influence processes (*subjective norms*, *voluntariness*, and *image*) and 4 cognitive processes (*job relevance*, *output quality*, *result demonstrability*, and *perceived ease of use*). A summary of how the full versions of TAM and TAM2 intersect is shown in Figure 1 (from Venkatesh and Davis [20]).

The additional features provided by the TAM2 enhancement are better suited for the contemporary, interconnected LAHP context of wide-ranging employee specializations. The adoption of TAM2 is also validated by similar recent studies using the framework, which is needed to accommodate similar complexity. This includes investigations into consumer perceptions of electronic health records in Australia [21], clinician adoption of internet-based health applications for pediatrics [22], and behavioral intentions of clinical staff to use radio frequency identification technology in hospitals [23].

**Figure 2:**
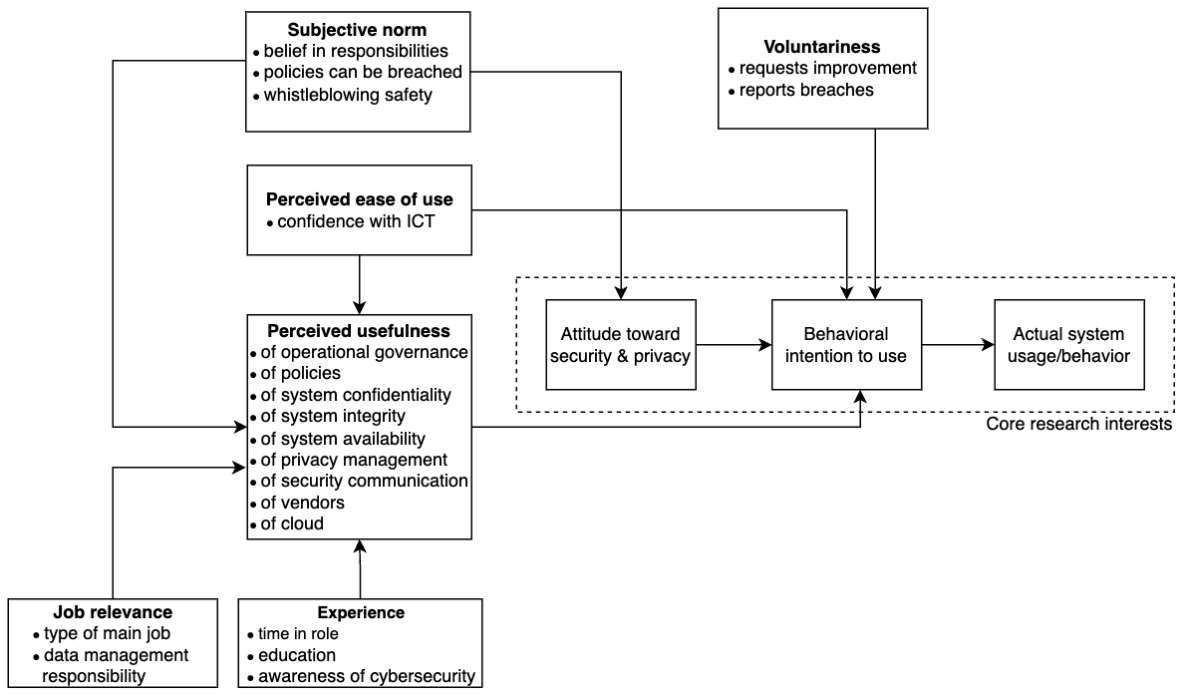
Modified Technology Acceptance Model (TAM2) motivations mapped to the variables used in this study. ICT: information and communication technology.

To support the consolidation of the findings from this study into verifiable conclusions, a final refinement of this model showing only the TAM2 motivations selected for this study was created. This includes the TAM2 title along with their relevant connections to the variables used in this research, as shown in Figure 2.

### Overall Research Design

In order to understand how sociological influences might impact security behaviors within heterogeneous LAHP staff populations, this study undertook a mixed methods study using an *explanatory sequential* design approach [24,25]. This comprised an initial quantitative survey, evaluated alongside a series of qualitative staff interviews. This methodology, anchored in Glaser and Strauss’ [26] grounded theory, was undertaken to facilitate a more complete set of findings via the empirically evidenced *reality*, and the phenomenological interpretations formed by individuals from varying professional backgrounds.

The importance of exploring these various aspects, rather than undertaking a singularly scientific-positivist path, is succinctly evidenced by Avorn [27], who wrote in *The* *Psychology of Clinical Decision Making*: “In reality, we [clinicians and patients] are all influenced by seemingly irrational preferences in making choices about reward, risk, time, and trade-offs that are quite different from what would be predicted by bloodless, if precise, quantitative calculations.”

The explanatory sequential approach was selected to feed-forward provisional findings from the quantitative survey instrument into a series of in-person qualitative interviews to discover and integrate details of their beliefs and motivations [24]. It is expected that this will help identify some of the irrationalities Avorn [27] indicates while also achieving the grounded theory goal of “discovering theory from data” [28]. This methodology was also selected to generate rationalized outcomes using the study’s integrated conclusions [29] in the process of data triangulation to explain both human and organizational complexities. This pragmatic focus was achieved via the (adapted) grounded theory proposed by Kesavan [30]:

- Stage 1: simultaneous collection and analysis of data
- Stage 2: a 2-step data coding process
- Stage 3: comparative methods
- Stage 4: memo writing aimed at the construction of conceptual analyses
- Stage 5: sampling to refine the author’s emerging theoretical ideas
- Stage 6: integration of the theoretical framework

**Figure 3:**
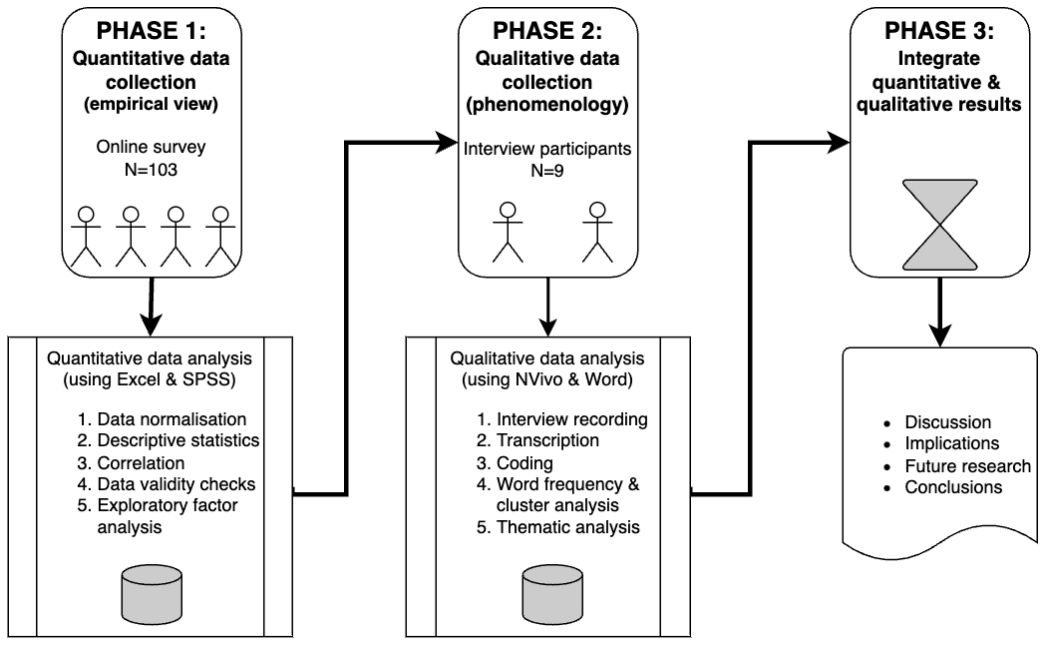
Model of the sequential explanatory mixed methods approach used in this paper.

The study design and data analysis undertaken in this study follow this process, with only the stage 4, *memo-writing* process, substituted with the notes produced to ultimately populate this paper. The workflow of the data collection and integrative evaluation process used in this study is summarized in Figure 3.

### Ethical Considerations

The study design and data collection approach for this research were submitted for human research ethics review board of Edith Cowan University, Australia, and was approved commencing April 30, 2020 (ref:2020-01418-DART). As part of the survey design, care was demonstrated to the ethics committee that protections were included regarding the informed consent, identity, and privacy of all participants, including the following controls (Multimedia Appendix 1):

- A participant information letter was supplied to all invitees for the interviews. It described the research process and provided university ethics and supervisory contacts.
- A participant consent form was provided to all invitees for the interview, confirming their permission to be recorded; however, their comments and identities would be protected and not further communicated without their explicit and informed consent.
- The invitation to participate in the web-based survey included an anonymous link to a Qualtrics (Qualtrics International Inc) hosted form, which included a shortened version of the participant information, including ethics approval and supervisor contact details.
- All users were advised of their right to withdraw from the research process at any time with no explanation required and with no penalty or other consequences.
- No payments or other inducements were available or suggested to any participants.

### Phase 1 (Survey) Methods

#### Overview

Phase 1 of this investigation (exploratory quantitative surveying) sought to achieve the outcomes of the survey research identified by Kraemer [31] (summarized in Textbox 1).

Given the intrinsic complexity across LAHP services and staff populations, the inferences referred to in stage 3 of Kraemer’s approach need some degree of subclassification or granularity. To achieve this, both the quantitative and qualitative phases used coding elements based on the theoretical framework of the extended TAM [19], known as TAM2 [20].

**The beneficial characteristics of survey research sought by this paper.**

**Kraemer characteristics and considerations or applicability to this paper**

- Survey research can quantitively describe aspects of a given population (including examining relationships among variables).
  This bespoke health care survey quantitively recorded variable aspects within the heterogeneous target population, so that formal correlations could be examined and analyzed.
- The data are gathered from people and therefore likely to be subjective.
  A wide range of people working in health care were invited to complete the survey, so that no single element of the staff population skews the results. This is reflective of the reality of staff operating within large health care systems.
  The survey also coded answers into single values for the purpose of quantitative correlation analysis (to seek meaningful relationships), allowing the mapping of opinions against other staff attributes.
- By using a selected portion of the population, reasonable inferences can extrapolated to the wider population.
  Again, a wide range of people (ages, experience, and career specialization) were invited to participate to adequately represent the typically heterogeneous status of a large health care system’s staff population.

#### Survey Design

A summary of the selected TAM2 motivations, encoded to relevant variable names and mapped against the questions presented to users via the survey, is shown in Table 1 (and fully expanded in Multimedia Appendix 2).

**Table 1:** Summary of Technology Acceptance Model 2 (TAM2) variables measured by questions in this survey.

TAM2 coding		Question number	Reason for inclusion or exclusion
**Included**			
	1. Job relevance (JR1, JR2)	1 and 4	To be able to evaluate behaviors or beliefs against one of the 3 tiers of job functions that are typically found within large health care systems or the degree of staff data management responsibility
	2. Experience (EX1, EX2, and EX3)	2, 3 and 7	To be able to evaluate each respondent’s results based on the duration in their role, educational level, or the existing awareness of cyber security issues in their profession
	3. Voluntariness (VO1 and VO2)	5 and 6	To establish if respondents had demonstrated previous behaviors in voluntarily seeking to improve their technology environment or reported what they perceived to be security incidents
	4. Perceived ease of use (PE1)	8	To establish which behaviors were related to an individual’s belief so that systems were easy for them to use
	5. Subjective norm (SN1, SN2, and SN3)	9 and 11e and 11h	To establish if respondents saw themselves as personally responsible for the security of clinical data and if they perceived whistleblowing or knowingly bypassing security as acceptable
	6. Perceived usefulness (PU1-10)	10 and 11a-11d, 11f, 11g, 11i, 11j	To establish respondent beliefs regarding which governance processes they considered most or least effective
**Excluded**			
	7. Output quality	N/A^a^	As details of cyber security outputs were not related to any of the roles being assessed via other variables, this measure was excluded. For this paper, the related measure of *perceived usefulness* of existing controls was sufficient
	8. Result demonstrability	N/A	Although this measure was excluded in the initial survey, it was proposed that via a subsequent survey process, the perception of cyber security outcomes positively impacting on health care job functions should be investigated
	9. Image	N/A	This measure was excluded in favor of measuring *subjective norms* for those behaviors that might be considered contentious (whistleblowing, willingness to breach policy, and individual responsibility). Further work on the perception of those actions on the individual would be of further interest, not prioritized in this survey

^a^N/A: not applicable.

#### Participant Selection and Sample Size

Defining an adequate survey sampling size can be problematic [32], particularly in a single-phase survey such as this, which sought to capture a large range of attributes over multiple questions. To meet the needs of this survey, the sample population therefore needed to be highly heterogeneous and randomized so that it effectively represented the employee population of a typical LAHP. In this regard, precision in the selection of respondents was less important than the holistic capture of attributes from each member of that population, with the main criterion being that the survey respondent was currently employed full time in an LAHP.

To minimize the possibility that respondents might consider the survey too time-consuming to complete, questions were presented in a simple webpage format (using the Qualtrics web-based survey platform), which was optimized to be readable on all mobile devices to facilitate convenience. Questions were authored to be as clear as possible without using technical terms or acronyms and did not require user registration or any training to complete. It was targeted to take between 10 and 15 minutes to complete. Two survey responses were left for 15 and 24 hours, respectively, between commencement and completion, and of the remaining 101 responses, the median time to complete was 6.2 minutes and the average was 8.21 (SD 7.52 min; 95% CI 6.72-9.69 min).

According to established social and information systems research which outlines that survey sample size needs to be “sufficient to support generalisations” [33], this survey sought to achieve a response rate above the minimum of 50 recommended by Taherdoost [34] and Van Voorhis et al [35]. The final completion rate was 103.

#### Data Collection

A randomized selection of participants was sought to represent the heterogeneity of staff working across a large health care system, stratified into three main groups: (1) patient-facing clinicians, (2) clinical support specialists, and (3) all forms of administrative and operational support. The survey was anonymous to attract the highest possible degree of engagement and to provide the highest standard of personal privacy to respondents in line with ethics approval.

An investigation was undertaken into the job role ratios of 25,798 health care employees, using figures published in the annual reports of 3 LAHPs. This returned averaged percentages of 55.16% (14,231/25,798) engaged as patient-facing staff, 25.05% (6463/25,798) in clinical support, and 19.78% (5104/25,798) in administrative or operational support roles. These became the target response rates for each stratum (independent variables) measured via the quantitative survey.

After ethics approval was granted, an initial email invite was sent to 1420 clinical staff members. This included a description of the research and a link to the Qualtrics web-based survey. A subsequent invite repeating this information was posted on an LAHP-based Yammer page (available to all staff) and on 4 Slack channels used by clinical and support staff (with approximately 80-100 users in each channel). Email invites were also provided to security managers at multiple LAHPs in all the states and territories of Australia, with a request for them to share via internal staff communication web pages. Finally, 30 additional users were e-mailed invites directly as part of the final convenience sample based on location and availability.

#### Analysis Techniques

Given that little other research exists in this area, it was important to thoroughly evaluate the quantitative data from these survey results, with the goal of ultimately undertaking an exploratory factor analysis (EFA). Therefore, six stages of review and verification were applied to validate the survey data and appraise the strength and indicative meaning of any relationship between the dependent (strata) variables and independent (beliefs and actions) variables examined [36]. This was achieved via the processes below using software tools including Microsoft V2301 (build 16.0.16026.20196; Microsoft Corporation), SPSS Statistics (V29.0.0.0; IBM Corp), and NVivo (12.6.1.970; QSR International).

#### Data Normalization

Five of the survey questions that provided respondents with >5 response options (qualifications, experience, data management, information and communication technology [ICT] confidence, and responsibility) were normalized to a scale of 1-5, using Microsoft Excel with the formula:

(5 – 1)*([x – MIN(x:*y*)] / [MAX(x: *y*) – MIN(x:*y*)]) + 1 **(1)**

This resulted in the final data set comprising 18 variables on a consistent 5-point scale and one retaining a 3-point nominal scale (JR1: job role). Another measure (PU2: preferred resourcing) also used a 5-point scale but was used only for a specific frequency analysis and was excluded from the correlation and EFA processes, as its content was distinctly subjective. A final examination of all responses showed that 6 surveys had missed recording an answer against one individual measure, and these were populated with 0 numerical values.

#### Descriptive Statistics

Response frequencies and percentages were captured across all survey measures and are reported in full in Multimedia Appendix 3, with the relevant measures analyzed in the results section.

#### Linear Consistency

Data distribution and linear consistency measures were applied to all 19 variables to identify any significant deviations that could distort the subsequent EFA process (the full output is detailed in Multimedia Appendix 4). These results show that while there are high (>+1) measures for skewness in the *improvements* and *breaches* variables (skewed right, stemming from low mean values), these are explained by the large number of survey respondents who reported no history of voluntary actions against either measure (70/103, 68% and 76/103, 73.8%, respectively). A slightly smaller left skew in *experience* was attributed to the large number of survey responses from more experienced staff members, with 70.9% (73/103) responding to the top 2 highest measures. The Kurtosis statistic measure (data distribution check) further confirms this phenomenon, showing sharp peaks in *improvements* and *breaches* due to the high single-score responses. None of the data showed an unexplained variance outside of these factors.

#### Cronbach α

With the data set normalized, Cronbach α was measured across 19 survey questions to generate a reliability coefficient for the variable set. Using the SPSS *reliability analysis* function configured to evaluate interitem correlations, a measure of α=.735 was obtained. This is within the *adequate* category of α≥.7 [37,38] and supports continued evaluation via subsequent statistical methods.

#### Bivariate Correlation

To provide an initial evaluation of empirical evidence against which to consider the phenomenology assessment stage undertaken in phase 2, a total of 19 of the quantitative survey question outcomes were processed via a bivariate correlation analysis using the SPSS software package (the output of this is shown in Multimedia Appendix 5). These correlations were sought to make *justified inferences* regarding existing beliefs and actions within the wider health care staff population [39] (hence, the one survey measure not evaluated here was the *preferred resourcing* question, which has little impact on staff behaviors on a daily basis).

The correlation weighting (*r*) and H_0_ test probability significance (*P*) measures used to evaluate these associations are treated per guidelines by Rosenthal [39] and are summarized in Table 2.

In the correlation results shown in Multimedia Appendix 5, Rosenthal’s [39] schema was used as the basis for highlighting (in bold text) only moderate correlations (*r*≥0.30; *P*≤.05) and significant correlations (*r*≥0.50; *P*≤.05). was evaluated with the creation of a Pearson coefficient matrix to measure the association (*r*) and significance (*P*) between all included variables.

**Table 2:** Baseline measures for r and *P* values used in this paper.

		Relationship	Interpretation
***r* (positive or negative correlation) values**			
	≈0.10	Small	Weak association
	≈0.30	Medium	Moderate association
	≈0.50	Large	Strong association
	≈0.70	Very large	Very strong association
***P* values**			
	≥.05	Weak or none	H_0_ is not rejected
	≥.02 but <.05	Average	H_0_ may be rejected
	≤.01	Strong	H_0_ is rejected

#### Exploratory Factor Analysis

Before commencing the EFA process, and in recognition that EFA is a process that has been extensively critically reviewed due to seemingly inconsistent researcher execution [40,41], a series of pretest evaluations were undertaken in addition to the measure of Cronbach α (.735) already established:

- Determinant score: a determinant score of 0.002 was reported for the data set, which was >0.00001, confirming that multicollinearity is not a concern [42] and that the EFA analysis can continue.
- Kaiser-Meyer-Olkin measure: a Kaiser-Meyer-Olkin calculation of all the variables selected was processed via SPSS to establish a value of *sampling adequacy* for each variable and the complete model [43]. With a generated measure of 0.741 (against the survey population of N=103), the total falls just short of the ideal*adequate* threshold of ≥0.8, but midrange within the *middling* scale (0.7-0.79), and well above the baseline of 0.6, indicating the need for remedial actions [43].
- Bartlett test of sphericity: the Bartlett test (*χ*^2^) was applied to evaluate the correlations previously generated and to establish if their relationships were strong enough to warrant subsequent EFA dimension-reducing processes. The analysis returned *χ*^2^=580.2 (*P*<.001), thereby rejecting the null hypothesis (“the variables are unrelated”) and confirming that the matrix is indeed nonorthogonal and sufficiently related to continue with the EFA process.

An EFA was then undertaken against the variable set to identify the clusters of potential influence on staff attitudes based on shared variance [44]. From this subset, the goal was to seek quantitative parsimony (the smallest number of explanatory concepts, applying a threshold of λ≥1.0) to explain the maximum amount of common variance across the analyzed variables [38]. The main factors identified via this process could then be examined alongside the qualitative interview outcomes to support thematic conclusions. The process outlined here for conducting the EFA largely follows the sample methodology outlined by Yong and Pearce [44], with further validation of measures and options from Watkins [38], Williams et al [45], and Shrestha [43].

**Figure 4:**
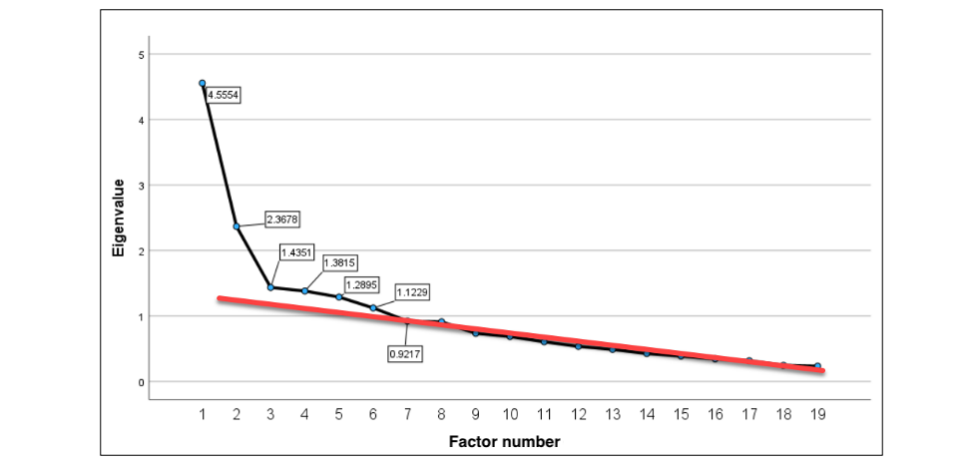
Scree plot (using prerotation eigenvalues), with the break line identifying significant (λ≥1) factors.

When running the EFA, additional configuration choices were configured as follows:

- Varimax was selected as the rotation method, which was confirmed after running the test rotations against 3 different methods (Varimax, Promax, and Oblimin). Although variable clustering within factors was very similar across each method, the non-Varimax methods both generated pattern matrix values >1 with no discernible reason, whereas Varimax returned all values <1. In addition, as this is an exploratory analysis, the Varimax attribute of tending to report a smaller range of important variables makes it more suitable to integrate findings via the mixed methods approach [46,47].
- The extraction method chosen was Principal Axis Factoring, so that weak factors from the relatively small sample size remained under consideration in the final output [48,49].
- Factor extraction was based on eigenvalues (λ) ≥1 and verified by applying the Scree Test method [45] illustrated in Figure 4 (showing 6 factors beyond the linearity *break line* linking the lower factors).

### Phase 2 (Interview) Methods

#### Overview

To seek confirmatory evidence of the findings emerging from the survey, a series of one-on-one interviews with health care staff was undertaken. These interviews were designed to further develop an etic (ie, outsider and specifically academic) understanding of the survey outcomes, informed by the emic (insider) narrative presented by the specialists interviewed [50,51]. Interview invitations were undertaken via purposeful sampling, with a deliberate attempt to interview staff members with differing professional expertise and experience. The adoption of these methods was intended to produce results toward what Emmel [52] summarizes as *“...*a descriptive unit that answers the question, often in considerable detail, what is going on here?”

Interviews were limited to 1 hour maximum and were either audio recorded in person or video recorded via web-based conference software. An intelligent verbatim transcription (using the techniques described by Eppich et al [53]) was made and imported into the NVivo qualitative analysis software tool, where coding and final analysis were completed.

Interviews were conducted in parallel with the survey data being captured and analyzed, and as provisional results from the survey emerged, they were used to prompt interviewees during a semistructured discussion. Nine employees from various departments and locations were interviewed; their summary characteristics are outlined in Table 3.

**Table 3:** Demographic summary of participants interviewed for this paper (N=9).

Code	Primary job role	Specialty area	Experience (years)
P1	Clinical practitioner and academic professor	Clinical delivery and developing health care digital solutions across Australia and internationally	30
P2	Clinical practitioner and academic professor	Clinical delivery and executive health care information management	20
P3	Nursing assistant	Nursing assignments across multiple facilities	11
P4	Manager	Health care contract and tender management	3
P5	Clinical practitioner and director	Emergency medicine and electronic records project management	10
P6	Clinical support technician and manager	Medical imaging across multiple sites	22
P7	Clinical practitioner and senior manager	Emergency medicine and systems governance	40
P8	Clinical practitioner and senior manager	Clinical care and clinical systems governance	25
P9	Cyber security professional	Information security, risk, and governance	4

#### Transcript Processing

The processing of all interview transcripts was undertaken in NVivo via multiple passes:

- Manuscripts were manually read after each interview, with the first-pass coding of the themes and ideas applied. The codes were aligned against the modified primary TAM2 headings identified earlier in this study, where obvious affiliation was present.
- A second read was undertaken, and coding details were completed across all transcripts based on the final modified TAM2 coding structure. Thematically based subcodes were added at this stage as required to capture the specific professional, personal, or cultural experiences reported by each staff member.

This coding structure and process were considered complete once thematic saturation appeared to have been achieved (ie, each of the transcripts had been read multiple times, and there were no apparent thematic gaps remaining or codes being applied).

During this process, text was coded according to the researchers’ subjective emic-etic *conversion* understanding, intended to capture the sentiment, context, or meaning spoken by each participant within each modified TAM2 primary category. This approach was undertaken to maintain the *truth* of the participants’ responses; explore potentially detailed correlations between each interview; and create a practical ontology that other researchers may subsequently interrogate, use in other research, or evaluate.

#### Word Frequency Analysis

A word frequency analysis was undertaken by combining quantitative and qualitative approaches in support of the grounded theory approach [15]. High levels of individual word frequencies (including closely related word derivatives, which are counted along with their *parent* word) are indicative that specific words, and their semantically associated topics, are of importance to specific groups of practitioners and should be recorded [54]. The methodology used to process all 9 interview transcripts for these analyses was undertaken in three stages:

1. Autogeneration of a word frequency analysis table using the NVivo built-in function to produce the top 100 (>3 characters) words in all transcripts, using interview participant answers only (interviewer questions and comments were excluded to prevent bias).
2. Manual checking of the table identified any irrelevant words, and these were added to the NVivo *Stop words* exclusion list and the word frequency analysis rerun.
3. When no further irrelevant words appeared, the table of the top 100 word occurrences was exported to Excel for the final formatting.

## Results

### Staff Survey: Descriptive Statistics

#### Demographics

The demographic strata applied via the survey’s first 3 questions showed that 54.4% (56/103) of the responses were gathered from staff engaged in patient-facing roles, 8.7% (9/103) were from clinical support, and 36.9% (38/103) from administrative or professional roles.

The largest job experience demographic group was those with >15 years of experience in their profession (58/103, 56.3%). This was followed by those with 10 to 15 years of experience (15/103, 14.6%) and 5 to 10 years (11/103, 10.7%). These 3 categories represented 81.6% (84/103) of all staff who responded.

Staff education levels were high, with 78.6% (81/103) holding either a bachelor’s degree, master’s degree, PhD, or other postgraduate qualifications.

#### Degree of Data Management Responsibility

We observed that 74.8% (77/103) of staff reported accessing patient data, with 60.2% (62/103) accessing administrative data, although across all staff a minority of 46.6% (48/103) reported that they wrote new or amended data as part of their everyday job. The smallest reported measure in this area was by staff who had been assigned formal data custodian duties (17/103, 16.5%).

#### Personal Security Behaviors and Comprehension Measures

The highest number of behavioral responses showed that most staff (70/103, 68%) had never volunteered any suggestions to improve the security or privacy of any LAHP system in the preceding 5 years. An even higher number (76/103, 73.8%) had never reported any form of data breach.

In terms of staff understanding how a data breach may present itself or impact systems, 34% (35/103) of staff had no knowledge of any data breaches impacting health care in the previous 5 years in any country at any time, while 42.8% (44/103) had awareness of only a few (1-5) such incidents.

#### Personal Beliefs or Opinions

Furthermore, 87.4% (90/102) of staff members believed that responsibility for the confidentiality, integrity, and availability (CIA) of clinical records is weighted more toward the health system, rather than the primary caregiver or clinician. Opinions were also captured regarding whom staff members considered best placed to manage future security improvements; 60.8% (62/102) of participants reported a preference for the health provider to resource an in-house security function, with 25.2% (26/102) believing that either the State or Federal government should provide this service. A minority of participants (3/102, 2.9%) believed that the private sector could meet this need.

The need for individuals to sometimes breach security or privacy policies to achieve optimal outcomes was disagreed or strongly disagreed with by 49.5% (51/103) of staff. The belief that risk and security best practices were effectively communicated to staff showed that 50.5% (52/101) either agreed or strongly agreed and that 22.3% (23/101) neither agreed nor disagreed.

Staff’s response to the opinion that the health provider is holistically managing security and privacy well for all stakeholders revealed 46.6% (48/103) agreeing or strongly agreeing, with 31.1% (32/103) unable to agree or disagree.

Staff views on the trustworthiness of hardware and software vendors in delivering a secure system recorded 45.6% (47/101) agreeing or strongly agreeing, and 23.8% (24/101) disagreeing or strongly disagreeing. A secondary question on the perceived trustworthiness of cloud computing became the only question where “neither agree nor disagree” was the largest response at 41.2% (42/102).

### Staff Survey: Correlation Results

A Pearson correlation coefficient matrix was computed to assess the linear relationships between the 19 surveyed variables. The full correlation matrix is shown in Multimedia Appendix 5, with the 9 pairs of positive associations identified as *strong*, detailed in Table 4.

**Table 4:** All positive large (r>0.50) and strong (*P*≤.01) variable correlations.

Variable #1	Variable #2	*r* value	*P* value
PU^a^7 (HolisticSecurity_Belief)	PU6 (Confidentiality_Belief)	0.650	<.001
PU6 (Confidentiality_Belief)	PU5 (Integrity_Belief)	0.605	<.001
PU7 (HolisticSecurity_Belief)	PU3 (Policy_Belief)	0.551	<.001
PU8 (Comms_Belief)	PU7 (HolisticSecurity_Belief)	0.534	<.001
PU4 (Availability_Belief)	PU3 (Policy_Belief)	0.529	<.001
PU7 (HolisticSecurity_Belief)	PU4 (Availability_Belief)	0.519	<.001
PU5 (Integrity_Belief)	PU4 (Availability_Belief)	0.518	<.001
PU8 (Comms_Belief)	PU5 (Integrity_Belief)	0.513	<.001
PU10 (Cloud_Belief)	PU9 (Vendors_Belief)	0.502	<.001

^a^PU: perceived usefulness.

### Staff Survey: EFA

After the data validation checks were completed, an EFA was executed via SPSS using an eigenvalue (λ) threshold of >1.0 (results shown in Table 5).

This analysis identified 5 factors that contributed to 43.6% of the cumulative variance across all measures. The sixth factor was dropped after rotation, as λ dropped from 1.12 to 0.74, reducing its significance.

**Table 5:** Factor loadings for all measures after rotation (loadings<0.5 suppressed).

	Factor evaluations^a,b^					
	*F*_1_: perceived usefulness (of systemic controls)	*F*_2_: perceived usefulness (of supply chain)	*F*_3_: experience (awareness via job exposure)	*F*_4_: job role (access to data)	*F*_5_: voluntariness (willing to speak up)	*F*_6_^c^: removed
Confidentiality_Belief (PU^d^6)	0.712	—^e^	—	—	—	—
Integrity_Belief (PU5)	0.690	—	—	—	—	—
HolisticSecurity_Belief (PU7)	0.679	—	—	—	—	—
Availability_Belief (PU4)	0.660	—	—	—	—	—
Policy_Belief (PU3)	0.622	0.333	—	—	—	—
Comms_Belief (PU8)	0.520	0.484	—	—	—	—
Vendors_Belief (PU9)	—	0.732	—	—	—	—
Cloud_Belief (PU10)	—	0.632	—	—	—	—
Whistleblowing_Belief (SN^f^3)	—	0.515	—	—	—	—
Awareness (EX^g^3)	—	—	0.646	—	—	—
Job_Role (JR^h^1)	—	—	0.621	—	—	—
Qualification (EX2)	—	—	0.507	—	—	—
Data_Management (JR2)	—	—	—	0.943	—	—
Experience (EX1)	—	—	—	—	—	—
Improvements (VO^i^1)	—	—	—	—	0.687	—
Breaches (VO2)	—	—	—	—	0.506	—
Breach_Belief (SN2)	—	—	—	—	—	—
Responsibility_Belief (SN1)	—	—	—	—	—	0.528
ICT_Confidence (PE^j^1)	—	—	—	—	—	—
λ (postrotation)	2.84	1.72	1.45	1.29	1.11	.74^a^
Variance (%)	14.9	9.1	7	6.8	5.8	3.9
Cumulative (%)	14.9	24	31	37.8	43.6	47.5

^a^Extraction Method: Principal Axis Factoring.

^b^Rotation Method: Varimax with Kaiser Normalization.

^c^Factor 6 excluded postrotation as λ<1.

^d^PU: perceived usefulness.

^e^Values <0.5 suppressed.

^f^SN: subjective norm.

^g^EX: experience.

^h^JR: job relevance.

^i^VO: voluntariness.

^j^PE: perceived ease of use.

### Interview Results

#### Transcript Coding

After input and analysis within NVivo, a total of 692 codes were applied across 9 interview transcripts, aligned to 5 primary TAM2 categories; 31 coded subthemes were applied to these TAM2 categories. The full details of code volumes assigned to each primary TAM2 and subcategory are presented in Multimedia Appendix 6.

In examining the coding applied across all interview transcripts, high volumes of TAM2-coded motivational drivers were identified within the *perceived usefulness* (281/692, 40.6%) and *subjective norms* (195/692, 28.2%) categories. The most frequently repeated individual codes within perceived usefulness included *risk*, *governance,* and *proposed solutions,* whereas the most common drivers from subjective norms were *people and relationships*, *patient confidentiality*, and *clinical exceptions* (to rules and policies).

#### Word Frequency Results

The top 100 most frequently occurring words mentioned by the interview participants (after excluding common or irrelevant words) are shown in Multimedia Appendix 7.

## Discussion

### Principal Findings

Using an explanatory sequential methodology, this study has shown via a quantitative analysis of survey data from 103 LAHP staff members that the *perceived usefulness* of security controls emerged as the most significant factor influencing their beliefs and behaviors (representing 24.03% of all variances). Through a further qualitative analysis of in-depth interviews with 9 staff members, issues of the *perceived usefulness* were also most frequently coded (281/692, 40.6%), followed by the *subjective norms* (195/692, 28.2%) resulting from the commonly adopted or witnessed behaviors of others. The word frequency analysis showed that *systems*, *patients,* and *people* represented the top 3 recurring themes reported by the interviewees.

Within these overall findings, there were multiple other indicators of interest that emerged, and these are explored in the following discussion in order of quantitative, qualitative, and combined implications.

### Data Management Responsibilities

In the daily management of data, understanding the role of staff was important for this paper to establish how much “skin in the game” they might have when it comes to measures of their normal behaviors and how relevant (or useful) they might consider security messaging to be. We noted that 74.8% (77/103) of staff reported that they access patient data for their job, which is a larger number than reported when they were employed in patient-facing roles (even when accommodating clinical support staff, this only equals65/103, 63.1%). This presents an important early observation, demonstrating that access to patient data is pervasive across many roles in an LAHP environment, outside of direct clinical care roles.

Most staff (62/103, 60.2%) also reported that they have access to or management responsibilities for administrative data, much of which may be essential to the operation of the wider health system (including ICT systems). However, it should be noted that the minority response in this category identifies that only 16.5% (17/103) of staff have been assigned formal data custodian responsibilities, suggesting that much of the management of important data repositories may be ad hoc or that management responsibilities are poorly understood.

### Personal Security Behaviors and Comprehension Measures

Understanding the prevalence and impact of security breaches on health care systems is an important element of gaining staff buy-in for improving security. This category of responses suggested that staff did not have this appreciation, with 76.8% (89/103) believing that there were none or very few such incidents. Given that in Australia, via figures reported by the Office of the Australian Information Commissioner, there have been 929 such incidents over the last 5 years [2], this is a concerning finding.

### Personal Beliefs or Opinions

This was the largest category of variables gathered, examining staff perceptions of *normal* or acceptable behaviors and their belief in the effectiveness of system security. One of the major findings from this area was that 87.4% (90/102) of staff reported that the CIA of clinical records was the responsibility of the health system, rather than the primary caregiver or clinician. Coupled with low levels of data custodianship reported (17/103, 16.5%), this perception has the potential to distort any concerns regarding responsibility and make it “someone else’s problem.”

A *preferred resourcing* question was included here to gauge the understanding of the future direction that staff would select to improve systemic governance around cyber security and privacy, such as commercial consultants, government-controlled centers, or health system–managed teams. Of note, 60.8% (62/102) reported a preference for the health system to manage this function themselves but reported a very small degree of support for commercial vendors to take on this role, with only 2.9% (3/102) believing that the private sector should or could meet this need. This measure has a further interesting aspect, given the *middle way* that emerged with 25.2% (26/102) identifying that either the national or state government should be operating such a function. This suggests that the highly experienced and educated staff in health care like to work within their own industry, and imposing security controls from monolithic government programs may not be well accommodated across all staff, leading to potentially fractured outcomes. This could also suggest that the best way to engage health care staff is to engage health care staff in delivering health care–specific messaging.

This measure was connected to staff views on the trustworthiness of hardware and software vendors to deliver secure systems, with 45.6% (47/101) agreeing or strongly agreeing and 23.3% (24/101) disagreeing or strongly disagreeing. When a similar question was framed around the trustworthiness of cloud computing, a more ambiguous picture emerged, with “neither agree nor disagree” as the largest response at 41.2% (42/102). These last 3 measures suggest that there is more work to be done in building trust with external entities and for health care staff to see themselves as part of a cyber frontline in the critical infrastructure space.

These findings are of some concern in Australia, as in recent years, the Commonwealth government has sought to incorporate health care explicitly via 2021 [55] and 2022 [56] amendments to the Security of Critical Infrastructure Act 2018 (Commonwealth) [57]. The results from the paper suggest that both government and health care leaders need to do more to help connect health care workers to those conversations and developments. Using the overall findings and recommendations from the paper is one means by which this might be approached with an improved chance of success.

### Correlation Analysis

The correlation analysis (detailed in Multimedia Appendix 5) shows that only one of the TAM2 *subjective norm* variables (SN2: Breach_Belief: “staff must sometimes breach existing systems security & data privacy policies”) lacked correlation with any other belief or behavior. This is an encouraging indication, which suggests that the staff belief in adhering to security policies is consistent across all specialties and that there were no pockets of staff noticeably willing to breach security controls.

### EFA Outcomes

The results of the EFA support the earlier observations of Pearson correlation, with *F*_1_ showing significant clustering around the TAM2 drivers of *perceived usefulness*, accounting for 14.9% (λ=2.84) of all variances. *F*_2_ shows a further 9.1% (λ=1.72) given to perceived usefulness (this time mostly of external vendors or cloud providers), whereas it is only at *F*_3_ that any element relevant to the job role (λ=1.45) comes into effect. Job role contributes again at *F*_4_ with 6.8% (λ=1.29) focused on data management responsibilities (which tend toward custodianship duties for more senior staff), and *F*_5_ completes the identified factors, capturing both forms of *voluntariness* in reporting measures (requesting privacy enhancements and reporting data breaches) at 5.8% (λ=1.11).

### Interview Coding

In examining the final data from the interview coding, themes assigned to the TAM2 variables via the survey data can be seen to align with comments from the interview participants. This allows the benefits of the explanatory sequential mixed methods approach to be realized, as examples of data triangulation emerge, showing similar outcomes and relationships, but from differing sources and perspectives.

In reviewing the application of thematic codes throughout the transcription review process, magnitudes of emotion and significance were evident as part of the interviewee’s emic interpretation of certain issues. To highlight the importance of these issues, the following sections show examples of the application of these primary and subtheme codes in their quoted contexts.

### Perceived Usefulness and Risk

Wide-ranging concerns related to risk management emerged from the perceived usefulness category, making it the most widely coded individual theme:

It came to my head that, I’m actually not insured either, so I rang up the Director, and he said “well, we’ve known you’re working, everyone tells us you’re hanging around.” And I laughed with him, and he said, “Oh well, we’d better fill in a form.” So, I filled in a form once, in two-and-a-half-decades of doing it. To cover off the theoretical liability.

If there’s something that can align that thinking of safety and security—would you report a safety near miss? Well, why wouldn’t you report an information loss near-miss, or security data security information near-miss?

Across the strata of staff specializations surveyed, the feedback was similar: there was frustration that existing risk assessments were not focused on practical risks and that nonclinical staff (bureaucrats) were making clinical staff undertake processes that were not aligned with issues of clinical or patient risk.

### Perceived Usefulness and Governance

A recurrent theme in the governance commentary was the inability of LAHPs to deliver sufficient large-scale governance capable of delivering the fundamental and systemic changes (which were especially important to the clinical staff strata):

The basic issue is, and I’ll give it to you in the strongest terms that I know how...I believe in the health department, there’s been a high-level failure of governance around digital services that goes back for at least 10 years.

The simple fact that we do not have an electronic medical record and we’re not even close. We’re not contemporary as a public health service, and that presents a clinical risk in terms of managing patients, particularly patients who are mobile and move around the state all the time. That’s our biggest governance failure.

### Perceived Usefulness: Proposed Solutions

Many examples were provided by all interview participants envisaging future improvements in technology, strategy, and policy. Again, it was the patient-facing staff who expressed frustration at current limitations while also displaying a willingness to consider new solutions:

A virtual environment, with rapid access in and out of that environment, would be a step in the right direction to solving the problems that I see.

So, radiology is an easy win. Telehealth is an easy win. In the country, you don’t have to have people travel hundreds of kilometres to talk to somebody for an hour—there’s lots of opportunities there. Where we aren’t really getting anywhere is on the floor in the hospital wards. How can we use technology to make that process more efficient?

**Perceived Usefulness: Policies**

References to policies were generally negative when clinicians reported them, with the “least negative” (perhaps best described as ambivalent) comments coming via the contract manager”:

Can I give you a tip (and this is a terrible thing to confess)? The vast majority of the policy that comes out of the Department IS shelfware.

You’re talking about what sort of policy, IT policy? I haven’t read it and I don’t know anyone who has.

From the cyber security professional interviewed, policy did not emerge any better:

Our primary Infosec Policy...what is it, about 15,16 pages long? I think there’s something wrong there. It shouldn't be that long. It shouldn't have that much detail.

These findings seem at odds with the survey data, which showed a strong specific correlation between belief in effective policies and a belief in the holistic security of systems (*r*=0.551; *P*<.001), and the factor analysis outcome showing *F*_1_ (representing 14.94% of all variances) comprised policy beliefs adjacent to confidentiality, integrity, and holistic security beliefs. The details that emerged from the interviews suggest that on a personal level, staff saw policies as limiting *their* freedom, but in a systemic sense, the fact that many policies exist lent those same staff to believe that they did contribute to overall security, but that *other* people (and the system) needed them. This is further supported by the survey data reported at SN1 (belief in custodianship), showing that most respondents (90/102, 87.4%) considered the CIA of clinical records to be the responsibility of the health system rather than the individual.

**Subjective Norms: People and Relationships**

Transcript analysis revealed a commonality of issues clustered around the TAM2 driver of *subjective norms* (with a significant component focused on the social influence inherent in personal and professional relationships). When discussing the behaviors, attitudes, and influence of people and relationships on security outcomes, the following quotes demonstrate recurrent staff motivators:

There is a community of Practice that gets engaged, and a variety of information sources that I engage in order to do the right thing for that patient.

I’m pretty sure there would be occasions when clinicians would send to other clinicians a photo, asking them for an opinion, and maybe even pictures of x-rays or something. But that’s principally because there is no good option for doing that in health systems, that are, you know, accessible for consulting with these people you are asking opinions of.

These themes highlight the perceived need to undertake data sharing or security actions, often in breach of LAHP policy, to participate in a broader community of practice that clinicians believe is to the ultimate benefit of the patient.

**Subjective Norms: Patient Confidentiality**

Regarding patient confidentiality (the second most common code associated with subjective norms), there was frequent agreement that practices were not ideal; however, due to the trust that exists between the clinical individuals involved in these bespoke processes, it was acceptable to participate in such deviations:

The world’s got a worse place because of the myriad of dodgy tools that we all have. You go back to the start of my career, it was far more secure, in patient data terms, when there was no mobile phones. I physically had to take the sheet of paper and walk round to my mate and say “well, what do you reckon?”

With junior doctors it doesn’t take long for a WhatsApp group to spring up. They might use initials and sometimes would talk about where the person is, so Mr. FG who’s in bed 4, but that’s risky because many people have the same initials.

In the survey responses, the reported trend was a positive belief in the CIA of data, but all the clinicians interviewed reported ready examples of data sharing, which were not confidential.

**Subjective Norms: Intersectionality**

An area arising out of the interviews relating to subjective norms, but which the survey did not directly query, was that of intersectionality in areas such as multiculturalism, income, and gender. In one case, an interviewee from an African background identified that staff who qualified and gained early career experience overseas might have quite a different outlook on legislative and social expectations regarding security, privacy, and governance that would otherwise be common in Australia:

In Africa there are not such strong privacy laws, and African staff will normally be less aware of privacy. There is not really a culture of personal privacy in Africa. This is why I do not choose to see an African doctor myself—I am worried they will Google me or ask about me in social situations I might see them in later. I have heard this from my friends.

A senior clinician described a similar theme, explaining how the culture within an Australian hospital would typically function around the personal relationships formed between colleagues who had graduated and worked together for many years:

As an ETS clinician, you act as a broker, particularly in rural areas where there is an itinerant workforce—everywhere from Africa to Melbourne, and they don’t know how to negotiate with the clinical community in the large teaching hospitals in [the city], so they’re attempting to refer someone who they think has a heart attack to a grumpy cardiology registrar in the city, and they will fail to do that due to communication or trust issues.

A further area of intersectionality that arose from one particular interview was that of wealth as a motivator for staff to even “care” very much about policy implementation:

I’ve done this for 30 years and you can’t control (clinicians), so you might as well fit in and work out how you can minimise the risk. The other story I share always is, and its back to that wealth problem, they don’t need to work for the health department. It’s almost an entertainment to them.

Neurosurgeons are a classic example. Their bread and butter is private practice, earning a quad-zillion dollars. Why then would they spend a day a week in the [hospital]? Because they get the one case in 4 million they otherwise never get to treat. They get to play with the widgets—the CT scanners or whatever—but it’s not about income. They are not employees in the sense of “I need to pay the mortgage.”

### Word Frequency Analysis

The word frequency analysis table shows that issues of “systems,” “patients” and “people” were most frequently mentioned by staff across all interviews. This indicates that staff concerns focused less on technical or security-specific issues and more on relationships, system workarounds, and effective service delivery. This is further evidenced by additional analysis of the top 100 words, showing that mentions of “security” only occur in sixth place, with “breach,” “password,” “technology,” and “login” all placed lower in the top 100.

### Actionable Insights for Health Care

An approach needs to be adopted in health care showing how good security is in fact an enabling prerequisite for the innovation many desire. It needs to be clearly communicated to staff that the delivery of very complex (and expensive) electronic medical record systems, which were mentioned 26 times across 6 interviews, is a pointless investment if they are quickly undermined by data breaches or failures resulting from poor user behaviors.

This study shows that this is not achieved by staff being force-fed training or dense security policies, but by ICT and security administrative staff recognizing the realities of clinical prioritizations and the culture of collaboration that prevails there. As such, it is important that security messaging is simplified and that a cultural shift is promoted across all areas. A recommended approach is to undertake the following 5-point approach to implement improvements:

1. Policies need to be reviewed, shortened, and combined with practical implementation advice. Creative writing and early, wide consultation are critical to this, as are options for distributing different language versions to staff from non–English-speaking backgrounds to assist with understanding. Policies that support, rather than penalize, the required channels for ad hoc clinician data sharing need to be created.
2. Training should be delivered as short, just-in-time messaging built into the host environment and workflow of staff members’ organizational settings.
3. Industry-standard security frameworks (ie, International Organization for Standardization 27001: information security management systems, or the National Institute of Standards and Technology Cyber Security Framework) need to be broken down and adapted to local use cases. Staged implementation should be based on collaborative service–focused risk assessments, and industry-relevant threat intelligence (ie, learning from incidents at other health care providers).
4. Security staff and architects should be involved in the early planning of strategic digital system replacements, to build trusted relationships with those deeply experienced and highly educated staff this research has identified are prevalent across many health care environments.
5. Security governance and operations need to be clearly developed as health care specializations, rather than tolerated only as external impositions based on audits or standards. Each LAHP should build a security team that can learn the priorities of service delivery and help integrate risk management, threat intelligence, and incident response processes into the patient care continuum.

The “hook” with which to help these actions succeed was illuminated through the word frequency analysis, with >100 mentions each for “system,” “patient,” and “people.” This exemplifies why a more inclusive, soft-systems approach that focuses on health care delivery effectiveness and people-focused outcomes is likely to be more effective. Staff who are attracted to the health care industry clearly care more about these issues than passwords, encryption, or multifactor authentication. The challenge, and clear opportunity that this research presents, is to reconcile and build connections between these interdependent concepts.

### Limitations, Contributions, and Future Work

Because of the immaturity of verified research into the social and behavioral influences on cyber security in large health care environments, this study had to consider a very broad scope of both potential influences and the cohort of staff from which to gather initial data. The limitations encountered included the relatively low rate of responses to the survey, given the volume of invitations sent, and the lack of granular detail obtained in understanding which intersectional subgroups each respondent might have associated with (due to a desire to keep the number of questions low). Gathering further context on these applicable staff subdimensions would also provide opportunities for further improving targeted messaging for staff using different techniques. Expanding the model, for example, by using the 6 sociological dimensions mentioned by Hofstede et al [14], is recommended for this exploration.

The mixed methods approach used in this study has proven to be highly effective in discovering and explaining the variability of existing security controls and behaviors within an LAHP. It has contributed to a useful 2-phase approach for quantitative and qualitative data gathering and has integrated them to produce practical insights for health care providers to adopt. The detailed validation of data for the EFA has presented a good example of how to conduct such an analysis for other research, and the coding of interview comments to the TAM2 variable has shown that such complex and unstructured data can be integrated using a mixed methods approach.

Using the same methodology to evaluate specific processes or information (such as training effectiveness, new policies, or enhanced systems) would be of great benefit in future work toward achieving pragmatic outcomes.

### Conclusions

In this study, we aimed to identify the beliefs and behaviors that influence the delivery of effective cyber security measures in LAHPs. Using an explanatory sequential mixed methods approach based on an adapted TAM2, this study has shown via both quantitative and qualitative means that *perceived usefulness* (of controls, outcomes, or actions) and the adoption of bespoke *subjective norms* emerged as the most significant factors influencing the heterogeneous staff cohort working in LAHP environments.

Previous research had theorized that sociological and nontechnical influences were likely to have a substantial impact on cyber security outcomes in health care; this study has provided specifics via both quantitative data measures and qualitative cross-correlations to confirm this.

As demonstrated in the interviews, staff reported particular frustration with policy documents that did not seem to have any practical outcome and an organizational approach that promoted investment in seemingly pointless security systems or ineffective legacy technology, as opposed to the emerging and innovative new clinical systems that many patient-facing staff have been demanding for many years.

This study further demonstrated that a solely mechanistic, or positivist approach, is unlikely to produce sufficient depth of results to explain or improve security outcomes in complex and relationship-dependent health care environments. Rather, a more systemic and multidisciplinary approach needs to be adopted that acknowledges and correlates the tacit and emic beliefs and behaviors developed by individuals. Subsequently, a more practical approach based on influence and persuasion, focusing on specific user communities, can steer those individuals to recognize and implement a different and improved approach to cyber security.

As has been demonstrated in both the quantitative and qualitative analyses, staff are more likely to improve their understanding and undertake more desirable cyber security behaviors if it can be demonstrated to them that the invested time and effort is of benefit in their everyday work practices. This is the *perception of usefulness* consistent with the TAM2 model and TPB identified as foundations for this research.

## Appendix

Multimedia Appendix 1 Participant information letter and consent form.

Multimedia Appendix 2 Full design and survey results.

Multimedia Appendix 3 Full survey results and descriptive statistics.

Multimedia Appendix 4 Table of data distribution and linear consistency checks.

Multimedia Appendix 5 Correlation analysis for all measures showing significant *P* values.

Multimedia Appendix 6 Coding statistics of all Technology Acceptance Model 2 themes across all interview transcripts.

Multimedia Appendix 7 Table of word frequency analysis results (top 100 words).

## Abbreviations

CIA: confidentiality, integrity, and availability
EFA: exploratory factor analysis
ICT: information and communication technology
LAHP: large Australian health care provider
TAM: Technology Acceptance Model
TPB: Theory of Planned Behavior

## Data Availability

All data that were generated or analyzed during this study are included in this manuscript (and its supplementary information files). Further deidentified data from the interviews are available from the corresponding author on demand.
