# Supplementary material for: Evaluating Staff Attitudes, Intentions, and Behaviors Related to Cyber Security in Large Australian Health Care Environments: Mixed Methods Study
Source: JMIR Hum Factors. 2023 Oct 4;10:e48220. doi: 10.2196/48220 (PMC10585427; doi:10.2196/48220)
Supplement: Multimedia Appendix 1 [file humanfactors_v10i1e48220_app1.pdf]

Chief Investigator: Martin Dart
Security Research Institute
Edith Cowan University
270 Joondalup Drive
JOONDALUP WA 6027
Phone: 0451 525 369
Email: m.dart@ecu.edu.au

Participant Information Letter

Project Title: Cybersecurity in public healthcare – developing a new approach
Approval Number: 2020-01418-DART
Principal Investigator: Martin Dart, supervised by Dr Mohi Ahmed

An invitation to participate in research

You are invited to participate in a project titled *Cybersecurity in public healthcare – developing a new approach,* which seeks to improve the management of cybersecurity risks within public sector healthcare environments. You are being asked to take part in this project because you have been identified as a stakeholder or participant in a relevant healthcare (or supporting) department. As such, we feel that you will be able to bring professional knowledge and practical experience to this research, that can help identify historic difficulties and evaluate potential new solutions.

This research project is being undertaken as part of the requirements of a PhD at Edith Cowan University.

Please read this information carefully. Ask questions about anything that you do not understand or want to know more about. Before deciding whether or not to take part, you might want to talk about it with a relative or friend.

If you decide you want to take part in the research project, you will be asked to sign the consent section. By signing it you are telling us that you:

- Understand what you have read;
- Consent to take part in the research project;
- Consent to be involved in the research described;
- Consent to the use of your personal information as described.

What is this project about?

This project aims to improve the management of cybersecurity risks within public sector healthcare environments. This will require gathering and analysing information on existing clinical workflows, corporate structures and policy, and evaluating their exposure to emerging threats from cybercrime. Proposals to improve these identified situations will then be scoped and evaluated.

What does my participation involve?

Your participation in this research may involve taking part in one or more of the following activities:

- **Completing an online questionnaire** - Duration 5 – 15 minutes per session.
- **One-to-one interviews** (in person or via telephone/teleconference) - regarding the issues identified in the survey. Duration 20 – 60 mins (max).
- **Group discussions** (in person, or via teleconference) - discussions and analysis of your actions, experience, understanding, or beliefs relating to information security management within your workplace, or related to your clinical treatment. Duration 1 – 2 hours (max).

This research is based on a methodology called *Action Research*, which consists of repeated stages of A: *Problem identification*; B: *Identification of possible solutions*; and C: *Evaluation of possible solutions (after implementation)*. As such your participation may be requested initially via one of the above activities to *define* a problem or possible solution, and you may then subsequently be asked to participate or comment via a different supporting process to *evaluate* a potential solution.

The in-person activities above will be audio-recorded electronically and subsequently transcribed into a de-identified digital record. Original recordings will then be securely deleted.

Do I have to take part in this research project?

Your participation in this research project is completely voluntary. If you do not wish to take part, you do not have to. If you decide to take part and later change your mind, you are free to withdraw from the project at any time.

If you do decide to take part, you will be given this Participant Information Letter and Consent form to sign and you will be given a copy of the information letter to keep. Your decision to take part, or to take part and later withdraw, will not affect your relationship with the research team and will have no relationship or bearing on your employment in any way.

Your privacy

By signing the consent form, you consent to the research team collecting your personal information for the purpose of participating in this research project. All such information obtained in connection with this research project that can identify you, including your opinions, comments, and contributions, will remain confidential.

At every stage your contributions and identity will be anonymised. This will be achieved by assigning you a generic participation identity in group discussions (i.e. 'participant #1', 'participant #2' etc); or when required identifying only your high-level role in the health system (i.e. 'clinical specialist #1', 'support director #2').

Your information and contributions will only be used for the purpose of this research project, and it will only be disclosed with your permission, except as required by law.

It is anticipated that the results of this research project will be published and/or presented in a variety of forums. In any publication and/or presentation, information will be provided in such a way that you cannot be identified, except where requested for specific reasons, and then you will be asked to provide a separate written consent.

In accordance with relevant Australian and/or Western Australian privacy and other relevant laws, you have the right to request access to the information about you that is collected and stored by the research team. You also have the right to request that any information with which you disagree be corrected. Please inform the research chief investigation identified at the top of this letter if you would like to access your information.

All data collected will be kept in accordance with ECU's Data Management Policy. Electronic data will be stored on servers housed at the ECU Security Research Institute which requires encrypted, multi-factor authentication to access. Physical records will be stored as required in ECU's Records Management Policy. The data will be retained for seven years, and destroyed, if appropriate at the end of the retention period under the State Records Act.

Possible Benefits

This research may not provide any immediate or personal benefit to you but may provide benefits for people working or being treated within the public service health system in the future.

Possible Risks and Risk Management Plan

There are no known risks to participating in this research project.

What happens when this research study stops?

All participants will be advised when the research has ended, and the outcomes accepted and published (either via Edith Cowan University, or relevant academic journals or conferences).

Your name or any other identifying information will not be included in any of the publications or presentations.

Has this research been approved?

This research project has received the approval of Edith Cowan University's Human Research Ethics Committee, in accordance with the National Health and Medical Research Council's *National Statement on Ethical Conduct in Human Research 2007 (Updated 2018)*. The approval number is 2020-01418-DART.

Contacts

If you would like to discuss any aspect of this project, please contact the following people.

Chief Investigator
Martin Dart
Edith Cowan University
P: 0451 525 369
E: m.dart@ecu.edu.au

Supervisor
Dr Mohi Ahmed
Edith Cowan University
P: +61 8 6304 5121
E: mohiuddin.ahmed@ecu.edu.au

If you have any concerns or complaints about the research project and wish to talk to an independent person, you may contact:

Research Ethics Support Officer
Edith Cowan University
P: 6304 2170
E: research.ethics@ecu.edu.au

If you wish to participate in this research, please sign the Consent Form and return to m.dart@ecu.edu.au.

Sincerely,

MARTIN DART

Chief Investigator

Chief Investigator: Martin Dart
Security Research Institute
Edith Cowan University
270 Joondalup Drive
JOONDALUP WA 6027
Phone: 0451 525 369
Email: m.dart@ecu.edu.au

Participant Consent Form

Project title: Cybersecurity in public healthcare – developing a new approach

Approval Number: 2020 – 01418 – DART

Principal Investigator: Martin Dart, supervised by Dr Mohi Ahmed

I, ___________________________________ have read the Participant Information Letter *or someone has read it to me in a language that I understand*. By signing this consent form, I acknowledge that I:

- have been provided with a copy of the Participant Information Letter, explaining the research study
- have read and understood the information provided
- have been given the opportunity to ask questions and have had questions answered to my satisfaction
- can contact the research team if I have any additional questions
- understand that participation in the research project may involve:
  - *In-person discussions and analysis of my actions, experience, understanding, or beliefs relating to information security management within my workplace, or related to my clinical treatment, either as a one-to-one interview or in a group setting. I understand and consent that this activity may be recorded in audio format.*
  - *Completing a questionnaire to record and analyse my activities, understanding, or beliefs relating to information security management activities within my workplace, or related to my clinical treatment.*
- understand that the information provided will be kept confidential, and that my identity will not be disclosed without consent
- understand that I am free to withdrawn from further participation at any time, without explanation or penalty
- freely agree to participate in the project

I agree to have my conversations audiotaped

Yes ☐ *No* ☐

Participant name: ____

Signature: ___________________________ Date ________________

Approval to conduct this research has been provided by the Edith Cowan University's Human Research Ethics Committee, approval number 2020 – 01418 – DART, in accordance with its ethics review and approval procedures.
