# Supplementary material for: Evaluating Staff Attitudes, Intentions, and Behaviors Related to Cyber Security in Large Australian Health Care Environments: Mixed Methods Study
Source: JMIR Hum Factors. 2023 Oct 4;10:e48220. doi: 10.2196/48220 (PMC10585427; doi:10.2196/48220)
Supplement: Multimedia Appendix 2 [file humanfactors_v10i1e48220_app2.pdf]

## Full design and coding of survey questions

| Category | Applicable TAM2 variable (and code) | Data validated response options (with coded values) | Method of measure |
|---|---|---|---|
| **QUANTITATIVE MEASURES** | | | |
| **Individual attributes** | Q1: Choose the option closest to your main job function in the health system: | | |
| | Job relevance (JR1) | (1) Administrative/Professional<br>(2) Clinical Support<br>(3) Patient-facing | Single answer permitted from multiple choice. |
| | Q2: How many years have you worked in your professional field? | | |
| | Job Experience (EX1) | (1) Less than 1 year<br>(2) 1-3 years<br>(3) 3-5 years<br>(4) 5-10 years<br>(5) 10-15 years<br>(6) 15+ years | Single answer permitted from multiple choice. |
| | Q3: What is your highest level of qualification in your professional field? | | |
| | Educational Experience (EX2) | (1) Certificate<br>(2) Diploma<br>(3) Bachelor's Degree<br>(4) Grad Diploma/Certificate<br>(5) Masters<br>(6) PhD | Single answer permitted from multiple choice *(Sourced from levels 1-5 of the Australian Standard Classification of Education (ASCED))* |
| **Degree of data management responsibility** | Q4: Select all options that apply to your role and management of data: | | |
| | Job relevance (JR2) | (1) Read data for purposes of job<br>(2) Access administrative data<br>(3) Write new or amended data<br>(4) Routinely access patient/clinical data<br>(5) Have formal custodianship duties | Multiple answers allowed (with coded values combined to create a total data management score (DMS)). |
| **Personal security behaviours & comprehension measures** | Q5: How many requests have you made to enhance the security or privacy of any healthcare information system in the last 5 years? | | |
| | Voluntariness (for suggesting/requesting improvements) (VO1) | (0) 0<br>(20) 1<br>(50) 2-5<br>(75) 5-10<br>(100) 10+ | Single answer permitted from multiple choice. |
| | Q6. How many times have you reported any inappropriate use or breach in the handling of official data in the last 5 years (by anyone)? | | |
| | Voluntariness (for reporting concerns) (VO2) | (0) 0<br>(20) 1<br>(50) 2-5<br>(75) 5-10<br>(100) 10+ | Single answer permitted from multiple choice. |
| | Q7: How many health sector cyber security attacks or unintended data breach incidents within healthcare are you aware of from the past 5 years (in any country/scale)? | | |
| | Experience (EX3) | (0) 0<br>(20) 1<br>(50) 2-5<br>(75) 5-10<br>(100) 10+ | Single answer permitted from multiple choice. |
| | Q8: Identify on the scale (0-10) your current self-assessment between the two statements: "When operating workplace ICT systems…" | | |
| | Perceived ease of use (confidence in the ICT system provided) (PE1) | (0) 0<br>(1) 1<br>(2) 2<br>(3) 3 | Single answer permitted from 11 bipolar matrix options, starting from 0, representing: |

| | | | |
|---|---|---|---|
| | | (4) 4<br>(5) 5<br>(6) 6<br>(7) 7<br>(8) 8<br>(9) 9<br>(10) 10 | *"I access only the basic functionality to achieve the outcomes I need. I worry about things going wrong I won't be able to fix."*<br>Through to 11, representing:<br>*"I like to explore advanced features & innovative use. If something doesn't work, I am confident to fix or configure it myself."* |
| **QUALITATIVE MEASURES** | | | |
| **Personal beliefs/ Opinions** | Q9: Choose your degree of preference between the 2 solutions to this statement: "Responsibility to maintain the confidentiality, availability and integrity of clinical records should reside with… | | |
| | Subjective norm (SN1) | (0) 0<br>(1) 1<br>(2) 2<br>(3) 3<br>(4) 4<br>(5) 5<br>(6) 6<br>(7) 7<br>(8) 8<br>(9) 9<br>(10) 10 | Single answer permitted from 11 bipolar matrix options, starting from 0, representing:<br>*"The Primary care giver or clinician"*<br>Through to 11, representing:<br>*"The Department of Health"* |
| | Q10: Based on the following statement, please place the following list into your ideal order of importance (starting with 1 as the most important). "To best ensure the security & privacy of healthcare systems & patient data across my Health system, the following resources should be prioritised" | | |
| | Perceived usefulness (of operational security governance) (PU1) | (1) A centralised Department of Health data & systems security operations centre<br>(2) Outsourced private sector security contracts<br>(3) An all-of-Government (state or national) security operations centre<br>(4) A clinician-focussed data & security advisory group<br>(5) Individual health service providers managing security at a local level | Ordinal ranking (1=highest preference) |
| | Q11: Please choose your preferred option in relation to each statement note 'Dept of Health' means the state-based entity you work for, even if it has a different name - it is NOT the Commonwealth/National Department of Health): | | |
| | Q11(a) The Dept of Health provides clear & effective system security and data privacy policies that supports clinical or business outcomes: | | |
| | Perceived usefulness (PU2) | (1) Strongly agree<br>(2) Agree<br>(3) Neither agree nor disagree<br>(4) Disagree<br>(5) Strongly disagree | Interval scale, from a matrix question, requiring a single answer from 5 choices. |
| | Q11(b) The current Dept of Health-provided information systems used in my field effectively maintains the AVAILABILITY of data: | | |
| | Perceived usefulness (PU3) | Scaled as at 11(a) above | |
| | Q11(c) The current Dept of Health-provided information systems used in my field effectively maintains the INTEGRITY of data: | | |
| | Perceived usefulness (PU4) | Scaled as at 11(a) above | |

| | | |
|---|---|---|
| | Q11(d) The current Dept of Health-provided information systems used in my field effectively maintains the CONFIDENTIALITY of data: | |
| | Perceived usefulness (PU5) | Scaled as at 11(a) above |
| | Q11(e) To deliver optimal outcomes, staff must sometimes breach existing Departmental systems security & data privacy policies: | |
| | Subjective norm (SN2) | Scaled as at 11(a) above |
| | Q11(f) The Dept of Health currently manages the holistic security and privacy needs for patients, clinicians & support staff effectively: | |
| | Perceived usefulness (PU6) | Scaled as at 11(a) above |
| | Q11(g) Information on emerging risks & best practice for the secure management of systems and patient data is effectively communicated to my work area by the Dept of Health: | |
| | Perceived usefulness (PU7) | Scaled as at 11(a) above |
| | Q11(h) If something went seriously wrong with a system or a patient's data while I was using it, I feel safe reporting it to the Dept of Health without blame being ascribed to me: | |
| | Subjective norm (SN3) | Scaled as at 11(a) above |
| | Q11(i) The vendors of clinical hardware or software systems can generally be trusted to deliver secure systems: | |
| | Perceived usefulness (PU8) | Scaled as at 11(a) above |
| | Q11(j) Cloud technology service providers can deliver clinically & professionally suitable services: | |
| | Perceived usefulness (PU9) | Scaled as at 11(a) above |
