# Supplementary material for: Evaluating Staff Attitudes, Intentions, and Behaviors Related to Cyber Security in Large Australian Health Care Environments: Mixed Methods Study
Source: JMIR Hum Factors. 2023 Oct 4;10:e48220. doi: 10.2196/48220 (PMC10585427; doi:10.2196/48220)
Supplement: Multimedia Appendix 3 [file humanfactors_v10i1e48220_app3.pdf]

Full descriptive survey results

A	B	C	D	E
**Category**	**Attribute**	**Total responses (ordered)**	**% per attribute**	**Cumulative %**
**1: Job function.**	Patient-Facing	56	54.4%	54.4%
(JR1)	Administrative/ Professional	38	36.9%	91.3%
(*n*=103)	Clinical Support	9	08.7%	100.0%
**2: Experience.**	15+	58	56.3%	56.3%
(EX1)	10 -15	15	14.6%	70.9%
(*n*=103)	5 – 10	11	10.7%	81.6%
	1 - 3	10	09.7%	91.3%
	3 - 5	6	05.8%	97.1%
	Less than 1	3	02.9%	100.0%
**3: Education.**	Bachelor's Degree	36	35.0%	35.0%
(EX2)	Master's degree	22	21.4%	56.4%
(*n*=103)	Grad Diploma/Certificate	19	18.4%	74.8%
	Certificate	12	11.7%	86.5%
	Diploma	10	09.7%	96.2%
	PhD	4	03.9%	100.0%
**4: Management of data.**	Access patient data (JR2.2)	77[a]	74.8%[b]	N/A
(JR2)	Read data	77	74.8%	N/A
(*n*=103)	Access admin data (JR2.1)	62	60.2%	N/A
	Write data	48	46.6%	N/A
	Data custodian (JR2.3)	17	16.5%	N/A
a. Multiple responses allowed. b. % of all respondents reporting this access (*n*=103)				
**5: No. of security/privacy enhancement requests made.**	0	70	68.0%	68.0%
(VO1)	2 – 5	16	15.5%	83.5%
(*n*=103)	10+	7	06.8%	90.3%
	1	7	06.8%	97.1%
	5 - 10	3	02.9%	100.0%
**6: No. of data breach reports made.**	0	76	73.8%	73.8%
(VO2)	1	11	10.7%	84.5%
(*n*=103)	2 – 5	10	09.7%	94.2%
	5 - 10	3	02.9%	97.1%
	10+	3	02.9%	100.0%
**7: Awareness of healthcare data breaches.**	0	35	34.0%	34.0%
(EX3)	2 – 5	29	28.2%	62.2%
(*n*=103)	1	15	14.6%	76.8%
	10+	15	14.6%	91.4%
	5 - 10	9	08.6%	100.0%
**8: Confidence Using Workplace ICT Systems**	6	20	19.4%	19.4%
(0=basic functionality: 11 explore advanced features)	8	13	12.6%	32.0%
(PE1)	7	12	11.7%	43.7%
(*n*=103)	11	10	09.7%	53.4%
	5	9	08.7%	62.1%
	4	8	07.8%	69.9%
	2	8	07.8%	77.7%
	9	8	07.8%	85.4%
	1	6	05.8%	91.3%
	3	6	05.8%	97.1%
	10	3	02.9%	100.0%
	0	0	00.0%	100.0%
**9: Belief in custodianship.**	6	44	43.1%	43.1%
(0=Clinician: 11 Health provider)	11	20	19.6%	62.7%
(SN1)	9	16	15.7%	78.4%
(*n*=102)	8	6	05.9%	84.3%
	4	4	03.9%	88.2%
	5	3	02.9%	91.2%
	7	3	02.9%	94.1%
	1	2	02.0%	96.1%
	3	2	02.0%	98.0%
	2	1	01.0%	99.0%
	10	1	01.0%	100.0%
	0	0	00.0%	100.0%

**10: Preferred Resourcing.**<br>(PU2)<br>(*n*=102)	Health Dept security centre	62	60.8%	60.8%
	Government security centre	26	25.5%	86.3%
	Clinician focused group	7	06.9%	93.1%
	Local health provider	4	03.9%	97.1%
	Private sector managed	3	02.9%	100.0%
**11(a): Clear & effective security policies provided.**<br>(PU3)<br>(*n*-103)	Agree	57	55.3%	55.3%
	Strongly Disagree	18	17.5%	72.8%
	Neither Agree nor Disagree	15	14.6%	87.4%
	Disagree	13	12.6%	100.0%
	Strongly Agree	0	00.0%	100.0%
**11(b) Data availability is effectively maintained.**<br>(PU4)<br>(*n*=103)	Agree	59	57.3%	57.3%
	Neither Agree nor Disagree	17	16.5%	73.8%
	Disagree	14	13.6%	87.4%
	Strongly Agree	10	09.7%	97.1%
	Strongly Disagree	3	02.9%	100.0%
**11(c) Data integrity is effectively maintained.**<br>(PU5)<br>(*n*=103)	Agree	53	51.5%	51.5%
	Neither Agree nor Disagree	24	23.3%	74.8%
	Strongly Agree	15	14.6%	89.3%
	Disagree	9	08.7%	98.1%
	Strongly Disagree	2	01.9%	100.0%
**11(d) Data confidentiality is effectively maintained.**<br>(PU6)<br>(*n*=103)	Agree	57	55.3%	55.3%
	Neither Agree nor Disagree	20	19.4%	74.8%
	Strongly Agree	15	14.6%	89.3%
	Disagree	10	09.7%	99.0%
	Strongly Disagree	1	01.0%	100.0%
**11(e) Staff must sometimes breach security & privacy policy.**<br>(SN2)<br>(*n*=103)	Disagree	30	29.1%	29.1%
	Agree	23	22.3%	51.5%
	Strongly Disagree	21	20.4%	71.8%
	Neither Agree nor Disagree	21	20.4%	92.2%
	Strongly Agree	8	07.8%	100.0%
**11(f) Holistic security & privacy needs are met by the health dept.**<br>(PU7)<br>(*n*=103)	Agree	35	34.0%	34.0%
	Neither Agree nor Disagree	32	31.1%	65.0%
	Disagree	21	20.4%	85.4%
	Strongly Agree	13	12.6%	98.1%
	Strongly Disagree	2	01.9%	100.0%
**11(g) Risk & best practice info is effectively communicated.**<br>(PU8)<br>(*n*=101)	Agree	44	43.6%	43.6%
	Neither Agree nor Disagree	23	22.8%	66.3%
	Disagree	20	19.8%	86.1%
	Strongly Agree	8	07.9%	94.1%
	Strongly Disagree	6	05.9%	100.0%
**11(h) I feel safe reporting incidents.**<br>(SN3)<br>(*n*=101)	Agree	48	47.5%	47.5%
	Strongly Agree	22	21.8%	69.3%
	Disagree	15	14.9%	84.2%
	Neither Agree nor Disagree	11	10.9%	95.0%
	Strongly Disagree	5	05.0%	100.0%
**11(i) Hardware and software vendors can be trusted.**<br>(PU9)<br>(*n*=101)	Agree	42	41.6%	41.6%
	Neither Agree nor Disagree	30	29.7%	71.3%
	Disagree	21	20.8%	92.1%
	Strongly Agree	5	5.0%	97.0%
	Strongly Disagree	3	03.0%	100.0%
**11(j) Cloud providers can deliver clinical outcomes.**<br>(PU10)<br>(*n*=102)	Neither Agree nor Disagree	42	41.2%	41.2%
	Agree	31	30.4%	71.6%
	Disagree	14	13.7%	85.3%
	Strongly Agree	11	10.8%	96.1%
	Strongly Disagree	4	03.9%	100.0%
