# Supplementary material for: Evaluating Staff Attitudes, Intentions, and Behaviors Related to Cyber Security in Large Australian Health Care Environments: Mixed Methods Study
Source: JMIR Hum Factors. 2023 Oct 4;10:e48220. doi: 10.2196/48220 (PMC10585427; doi:10.2196/48220)
Supplement: Multimedia Appendix 4 [file humanfactors_v10i1e48220_app4.pdf]

Data Distribution & Consistency Statistics

	N	Minimum	Maximum	Mean	Std. Deviation	Skewness		Kurtosis	
	Statistic	Statistic	Statistic	Statistic	Statistic	Statistic	Std. Error	Statistic	Std. Error
Job_Role	103	1	3	1.83	.944	.359	.238	-1.801	.472
Qualification	103	1	5	2.96	.969	-.448	.238	.216	.472
Experience	103	1	5	4.12	1.174	-1.046	.238	-.106	.472
Data_Management	103	1	5	2.93	1.012	.254	.238	-.820	.472
Improvements	103	1	5	1.74	1.228	1.520	.238	1.168	.472
Breaches	103	1	5	1.50	.989	2.064	.238	3.646	.472
Awareness	103	1	5	2.55	1.412	.411	.238	-1.043	.472
ICT_Confidence	103	1	5	3.05	1.183	-.132	.238	-.600	.472
Responsibility_Belief	103	1	5	3.50	.979	-.014	.238	-.164	.472
Policy_Belief	103	1	4	2.22	.885	.669	.238	-.115	.472
Availability_Belief	103	1	5	2.43	.946	.888	.238	.322	.472
Integrity_Belief	103	1	5	2.32	.899	.721	.238	.508	.472
Confidentiality_Belief	103	1	5	2.27	.865	.735	.238	.428	.472
Breach_Belief	103	1	5	3.32	1.246	-.231	.238	-1.040	.472
HolisticSecurity_Belief	103	1	5	2.65	1.007	.106	.238	-.725	.472
Comms_Belief	103	0	5	2.67	1.115	.254	.238	-.314	.472
Whistleblowing_Belief	103	0	5	2.29	1.160	.676	.238	-.125	.472
Vendors_Belief	103	0	5	2.70	1.008	.053	.238	-.047	.472
Cloud_Belief	103	0	5	2.67	1.004	.054	.238	.032	.472
Valid N (listwise)	103								
