# Supplementary material for: Evaluating Staff Attitudes, Intentions, and Behaviors Related to Cyber Security in Large Australian Health Care Environments: Mixed Methods Study
Source: JMIR Hum Factors. 2023 Oct 4;10:e48220. doi: 10.2196/48220 (PMC10585427; doi:10.2196/48220)
Supplement: Multimedia Appendix 5 [file humanfactors_v10i1e48220_app5.pdf]

Pearsons Correlation Table																					
			1	2	3	4	5	6	7	8	9	10	11	12	13	14	15	16	17	18	19
1. Job_Role	JR1	Pearson Correlation	1																		
2. Qualification	EX1	Pearson Correlation	**.325****	1																	
		Sig. (2-tailed)	**<.001**																		
3. Experience	EX2	Pearson Correlation	.019	.142	1																
		Sig. (2-tailed)	.852	.153																	
4. Data_Management	JR2	Pearson Correlation	-.177	-.053	.295**	1															
		Sig. (2-tailed)	.074	.597	.002																
5. Improvements	VO1	Pearson Correlation	.104	.049	.178	-.078	1														
		Sig. (2-tailed)	.296	.623	.072	.436															
6. Breaches	VO2	Pearson Correlation	.232*	.143	.270**	.133	**.328****	1													
		Sig. (2-tailed)	.018	.148	.006	.182	**<.001**														
7. Awareness	EX3	Pearson Correlation	**.382****	**.352****	.185	.102	.226*	**.437****	1												
		Sig. (2-tailed)	**<.001**	**<.001**	.061	.305	.022	**<.001**													
8. ICT_Confidence	EX4	Pearson Correlation	.218*	.044	.024	.199*	.204*	.188	.265**	1											
		Sig. (2-tailed)	.027	.656	.809	.044	.038	.057	.007												
9. Responsibility_Belief	SN1	Pearson Correlation	.054	.073	-.069	.025	-.068	.048	-.154	.224*	1										
		Sig. (2-tailed)	.588	.467	.490	.802	.493	.630	.119	.023											
10. Policy_Belief	PU3	Pearson Correlation	.200*	.102	.211*	.138	.000	.206*	.143	.083	-.064	1									
		Sig. (2-tailed)	.043	.307	.033	.166	.998	.037	.149	.403	.524										
11. Availability_Belief	PU4	Pearson Correlation	.172	.232*	.184	.154	-.055	.270**	.152	.016	-.055	**.529****	1								
		Sig. (2-tailed)	.082	.018	.062	.122	.584	.006	.126	.870	.580	**<.001**									
12. Integrity_Belief	PU5	Pearson Correlation	.101	.138	.178	-.008	.050	.279**	.183	.068	-.119	**.451****	**.518****	1							
		Sig. (2-tailed)	.309	.164	.072	.935	.615	.004	.064	.494	.232	**<.001**	**<.001**								
13. Confidentiality_Belief	PU6	Pearson Correlation	.143	.176	.133	.111	-.006	.182	**.309****	-.023	-.221*	**.432****	**.420****	**.605****	1						
		Sig. (2-tailed)	.150	.075	.182	.265	.951	.066	**.002**	.821	.025	**<.001**	**<.001**	**<.001**							
14. Breach_Belief	SN2	Pearson Correlation	.031	.027	.075	-.021	-.188	-.228*	-.091	-.057	-.013	-.101	-.067	-.093	-.154	1					
		Sig. (2-tailed)	.753	.789	.453	.830	.057	.021	.363	.566	.894	.310	.499	.353	.120						
15. HolisticSecurity_Belief	PU7	Pearson Correlation	.183	.267**	.275**	**.303****	.004	.287**	**.337****	.031	-.157	**.551****	**.519****	**.493****	**.650****	-.144	1				
		Sig. (2-tailed)	.065	.006	.005	**.002**	.964	.003	**<.001**	.757	.112	**<.001**	**<.001**	**<.001**	**<.001**	.146					
16. Comms_Belief	PU8	Pearson Correlation	.047	.242*	.284**	**.380****	-.100	.090	.086	-.062	-.133	**.513****	**.470****	**.361****	**.348****	-.121	**.534****	1			
		Sig. (2-tailed)	.636	.014	.004	**<.001**	.317	.364	.388	.533	.180	**<.001**	**<.001**	**<.001**	**<.001**	.225	**<.001**				
17. Whistleblowing_Belief	SN3	Pearson Correlation	-.016	-.051	.047	.050	-.152	-.121	-.081	-.060	.051	.289**	.145	.173	.125	-.065	.197*	**.401****	1		
		Sig. (2-tailed)	.875	.610	.639	.613	.125	.224	.414	.544	.612	.003	.145	.081	.207	.513	.046	**<.001**			
18, Vendors_Belief	PU9	Pearson Correlation	.109	.179	.220*	.018	.007	.036	.104	-.029	-.113	**.362****	.270**	.270**	.230*	.031	**.359****	**.443****	**.394****	1	
		Sig. (2-tailed)	.273	.071	.025	.855	.945	.719	.294	.773	.257	**<.001**	.006	.006	.020	.758	**<.001**	**<.001**	**<.001**		
19. Cloud_Belief	PU10	Pearson Correlation	-.103	-.023	.149	.113	-.198*	-.048	.019	-.151	-.128	.216*	.078	.097	.138	.211*	.292**	**.340****	**.328****	**.502****	1
		Sig. (2-tailed)	.301	.815	.132	.257	.045	.632	.845	.127	.197	.028	.435	.332	.164	.033	.003	**<.001**	**<.001**	**<.001**	

**. Correlation is significant at the .01 level (2-tailed).
*. Correlation is significant at the .05 level (2-tailed).
