# Supplementary material for: Evaluating Staff Attitudes, Intentions, and Behaviors Related to Cyber Security in Large Australian Health Care Environments: Mixed Methods Study
Source: JMIR Hum Factors. 2023 Oct 4;10:e48220. doi: 10.2196/48220 (PMC10585427; doi:10.2196/48220)
Supplement: Multimedia Appendix 6 [file humanfactors_v10i1e48220_app6.pdf]

| TAM2 + sub-theme identifier | Notes | Coded in (*x*) manuscripts | Code Totals (*n*) | Theme Totals (*x*) |
|---|---|---|---|---|
| **1. Experience** | Career experience (in healthcare) | 7 | 20 | |
| 1.1. Security stories | Examples of workarounds/experiences | 7 | 41 | |
| 1.2. Data breaches | Via insider threat or external attacks | 6 | 26 | |
| 1.3. Awareness of incidents/breaches | Within own workplace or elsewhere | 6 | 24 | 111 |
| **2. Perceived Ease of Use** | Support security delivery to services | 1 | 1 | |
| 2.1. Mobile devices | Phones/personal devices in workplace | 6 | 25 | |
| 2.2. Passwords & ID management | Identity challenges (inc. SSO) | 6 | 21 | |
| 2.3. New & innovative systems | For delivering better healthcare | 5 | 16 | |
| 2.4. Mobile working | Working across multiple sites | 4 | 12 | |
| 2.5. Oversight/supervision | Assistance with new tech/policy | 6 | 8 | 83 |
| **3. Perceived Usefulness** | Contribution to good service outcomes | 1 | 1 | |
| 3.1. Risk | To patient health, data, or systems | 8 | 53 | |
| 3.2. Governance | Of security or overall digital demand. | 7 | 37 | |
| 3.2.1. KPIs | Performance indicators for security | 3 | 4 | |
| 3.3. Proposed solutions | To improve uptake of security | 6 | 33 | |
| 3.4. Policies | Existing or proposed | 8 | 29 | |
| 3.5. Patient outcomes | Effectiveness of healthcare service | 7 | 26 | |
| 3.6. Electronic Medical Records | Use of emerging large digital systems | 6 | 26 | |
| 3.7. Vendors & partners | Inc. Public Private Partnerships | 6 | 26 | |
| 3.7.1 Cloud computing | Resource delivery/ data sovereignty | 3 | 7 | |
| 3.8. Accreditation & standards | For clinical services | 3 | 14 | |
| 3.9. Training | In any aspect of digital security | 7 | 13 | |
| 3.10. Legacy systems | For health or ICT service delivery | 3 | 9 | |
| 3.11. Clinical coding | Recording of clinical activities | 3 | 5 | 281 |
| **4. Subjective Norms** | What staff experience in workplace | 0 | 0 | |
| 4.1. People & relationships | Exploitation of personal relationships | 7 | 48 | |
| 4.2. Patient confidentiality | Examples of good/bad practice | 7 | 41 | |
| 4.3. Clinical exceptions | Reasons clinicians breach cyber policy | 7 | 28 | |
| 4.4. Motivation | Improving security via self-interests | 6 | 20 | |
| 4.5. Paper records | References to the use of paper records | 8 | 20 | |
| 4.6. Social media use | Inappropriate sharing of clinical data | 4 | 11 | |
| 4.7. Contracts | With commercial entities | 1 | 10 | |
| 4.8. Intersectionality | Identification of staff sub-groupings | 5 | 9 | |
| 4.9. Multiculturalism | Of the workforce or patients | 6 | 8 | |
| 4.10. Monitoring content use | Governance oversight of system use | 2 | 2 | 195 |
| **5. Voluntariness** | Willingness to take initiative action | 1 | 1 | |
| 5.1. Reporting incidents, breaches | Whistleblowing or raising concerns | 8 | 16 | |
| 5.2. Making improvements | Taking action when needed | 2 | 5 | 22 |
