# Supplementary material for: Evaluating Staff Attitudes, Intentions, and Behaviors Related to Cyber Security in Large Australian Health Care Environments: Mixed Methods Study
Source: JMIR Hum Factors. 2023 Oct 4;10:e48220. doi: 10.2196/48220 (PMC10585427; doi:10.2196/48220)
Supplement: Multimedia Appendix 7 [file humanfactors_v10i1e48220_app7.pdf]

Rank	Word	Freq	Weighted (%)	Rank	Word	Freq	Weighted (%)
1	System	135	1.7	51	Aware	18	0.2
2	Patient	115	1.5	52	Confidentiality	18	0.2
3	People	109	1.4	53	Documents	18	0.2
4	Information	81	1.0	54	Model	18	0.2
5	Health	69	0.9	55	Moving	18	0.2
6	Security	62	0.8	56	Reason	18	0.2
7	Hospital	56	0.7	57	Standards	18	0.2
8	Need	56	0.7	58	Device	17	0.2
9	Record	55	0.7	59	Environment	17	0.2
10	Imaging	51	0.7	60	Phone	17	0.2
11	Shared	49	0.6	61	Report	17	0.2
12	Clinicians	48	0.6	62	Messaging	16	0.2
13	Access	45	0.6	63	Solution	16	0.2
14	Data	42	0.5	64	Help	16	0.2
15	Issue	41	0.5	65	Nurses	15	0.2
16	Private	39	0.5	66	Level	15	0.2
17	Clinically	38	0.5	67	Site	15	0.2
18	Policy	38	0.5	68	Electronic	14	0.2
19	Providers	36	0.5	69	Example	14	0.2
20	Person	33	0.4	70	Recently	14	0.2
21	Medical	33	0.4	71	Mates	14	0.2
22	Care	32	0.4	72	Notes	14	0.2
23	New	32	0.4	73	PACS	14	0.2
24	Send	32	0.4	74	Paper	14	0.2
25	Managing	30	0.4	75	Password	14	0.2
26	Different	30	0.4	76	Pay	14	0.2
27	Taking	29	0.4	77	States	14	0.2
28	Terms	27	0.4	78	Involved	13	0.2
29	Staff	26	0.3	79	Better	13	0.2
30	Problems	26	0.3	80	Everyone	13	0.2
31	Service	26	0.3	81	Improve	13	0.2
32	Departments	25	0.3	82	Safety	13	0.2
33	Forms	25	0.3	83	Technology	13	0.2
34	Community	24	0.3	84	WhatsApp	13	0.2
35	Fax	24	0.3	85	Certainly	13	0.2
36	Places	23	0.3	86	Control	13	0.2
37	Something	23	0.3	87	Login	13	0.2
38	Trying	23	0.3	88	Results	13	0.2
39	Call	22	0.3	89	Running	13	0.2
40	Support	22	0.3	90	Ward	13	0.2
41	Doctors	21	0.3	91	Important	12	0.2
42	Local	21	0.3	92	Able	12	0.2
43	Network	21	0.3	93	Interact	12	0.2
44	Privacy	21	0.3	94	Kind	12	0.2
45	Redacted	21	0.3	95	Looking	12	0.2
46	Breach	20	0.3	96	Old	12	0.2
47	Digital	20	0.3	97	Process	12	0.2
48	Contracts	20	0.3	98	Change	11	0.1
49	Team	19	0.3	99	Computers	11	0.1
50	Understand	19	0.3	100	Correct	11	0.1
